# Learning and Exposure Affect Environmental Perception Less than Evolutionary Navigation Costs

**DOI:** 10.1371/journal.pone.0059690

**Published:** 2013-04-05

**Authors:** Russell E. Jackson, Chéla R. Willey, Lawrence K. Cormack

**Affiliations:** 1 Psychology and Communication Studies Department, University of Idaho, Moscow, Idaho, United States of America; 2 Psychology Department, University of California Los Angeles, Los Angeles, California, United States of America; 3 Psychology Department, University of Texas, Austin, Texas, United States of America; Monash University, Australia

## Abstract

Most behaviors are conditional upon successful navigation of the environment, which depends upon distance perception learned over repeated trials. Unfortunately, we understand little about how learning affects distance perception–especially in the most common human navigational scenario, that of adult navigation in familiar environments. Further, dominant theories predict mutually exclusive effects of learning on distance perception, especially when the risks or costs of navigation differ. We tested these competing predictions in four experiments in which we also presented evolutionarily relevant navigation costs. Methods included within- and between-subjects comparisons and longitudinal designs in laboratory and real-world settings. Data suggested that adult distance estimation rapidly reflects evolutionarily relevant navigation costs and repeated exposure does little to change this. Human distance perception may have evolved to reflect navigation costs quickly and reliably in order to provide a stable signal to other behaviors and with little regard for objective accuracy.

## Introduction

Does experience change how we see the physical world? For example, do regularly traveled paths look differently to us than they did when we first encountered them? Can repeated exposure change that which we perceive about our environment, or is perception fixed in place by the time we reach adulthood? Navigation of the environment is prerequisite to most behaviors and so the effects of learning on environmental perception are fundamental for understanding the precursors to most human and non-human animal behavior.

It is easy to determine the effects of experience on navigation: merely measure perceived distance across repeated exposure. Unfortunately, such research (see below) is rare and has yet to address this phenomenon in adults clearly. Adult navigation of familiar environments likely comprises the vast majority of human navigation, yet we have little understanding of how or if this changes over time. Further, the primary research approaches investigating distance perception and learning set forth mutually exclusive predictions (see below).

The key to understanding how we perceive common distances may lie in understanding the risks and costs associated with navigation. Recent theoretical advances have identified previously unknown navigational and perceptual mechanisms by focusing on the evolutionarily relevant costs of navigation [1], [2]. However, researchers have not yet applied these theoretical advances to address the potential effects of repeated exposure.

Below, we compare predictions from three major research approaches that investigate the effects of repeated exposure on distance perception. We outline predictive differences in 1) the extent to which, and direction in which, repeated exposure alters distance perception, and 2) the extent to which distance perception reflects evolutionarily relevant navigation costs and risks. The subsequent experiments then test these predictions in individuals with different levels of exposure on everyday surfaces such as stairs and flat ground.

### Retinal Image Hypothesis

A common technique for understanding distance perception within the vision sciences focuses on retinal image. Visual, especially distance, perception may be determined nearly entirely by the way in which images fall on the retina (occasionally referred to as the Law of Retinal Image [3]). An important feature of this approach is that the amount of space occupied by an image on the retina roughly determines the size of its corresponding object in the real world. For example, distances that are perpendicular to the line of sight occupy more space on the retina, and appear longer, than equal distances that are parallel to the line of sight [4], [5]. Further, observers tend to perceive equal distance from lines of similar orientation [6].

Under this hypothesis, distance perception is primarily a product of the amount of space occupied on the retina, which is constant across repeated exposure. Exposure, beyond a brief initial opportunity to perceive a surface, should not change the distance perceived from it.

Additionally, navigation risks should not alter distance perception under this hypothesis. The potential risks of navigating a surface do not alter the image that it casts on the retina in a universal fashion. For example, a sloped surface that poses a risk of falling occupies more space on the retina when the observer stands at the bottom of it than it does when the observer stands at its top; this is because the slope appears nearly perpendicular to the line of sight while standing at its bottom, but its angle is nearly the same as the line of sight while standing at its top. In fact, the image of a downward sloping surface (tested in Experiments 2 through 4 below) occupies less area on the retina than a similar horizontal surface and should thus appear shorter than the horizontal surface under this hypothesis.

#### Retinal image prediction 1

Distance perception should not substantially change across different levels of exposure to a surface.

#### Retinal image prediction 2

Distance perception should not substantially change across different levels of risk or cost in navigating the viewed surface.

Both predictions assume that the retinal image is roughly equivalent across different levels of exposure or navigation risk. If the retinal image changes across conditions, then we should predict that perception of surface length will either decrease or increase, respectively, as the image of the surface occupies less or more space on the retina.

### Learning Hypothesis

Substantial learning research proposes that distance estimation does become increasingly accurate with repeated exposure [6]–[12]. Interestingly, although some designed learning experiences may improve one’s ability to execute specific distance *estimation* procedures [13], [14], there is little clear evidence of increased accuracy in the underlying *perception* via passive exposure, certainly in adults. Whether or not exposure actually alters adult distance perception remains unclear, despite substantial research conducted under this premise.

Concerning navigation costs, the effects of learning should not be limited to one specific level of navigation cost or risk. We should learn greater distance estimation accuracy with greater exposure to a surface–whether or not the surface is risky to navigate. Humans should perceive distances increasingly accurately across repeated exposure and across levels of navigation risk.

#### Learning prediction 1

Distance perception should become increasingly accurate at greater levels of exposure to a surface.

#### Learning prediction 2

Distance perception should not substantially change across different levels of risk or cost in navigating the viewed surface.

### Evolved Navigation Theory

Evolved navigation theory (ENT) is a relatively recent research approach that focuses on how the costs of navigation over evolutionary time may have shaped navigational, perceptual, or locomotor processes [1], [15]. One component suggested under ENT is that distance perception may reflect navigational costs or risks more than it reflects objective accuracy. Humans navigate longer (perceived) distances less than shorter (perceived) distances, likely because organisms tend to pursue the nearest of otherwise equivalent distances [16], [17].

Predictions derived from ENT have led to the discovery of some of the largest distance illusions known in everyday vision. Vertical surfaces pose a navigation risk of falling unequalled by the costs of navigating horizontal surfaces. In response, ENT researchers predicted and then discovered the *environmental vertical illusion*, in which humans estimate environmentally vertical surfaces as longer than equivalent horizontal surfaces by up to 51% [18]. Further, descent of a vertical surface poses greater likelihood, and costs, of falling than ascending the same surface. In response, ENT researchers predicted and then discovered the *descent illusion*, in which observers overestimate a vertical surface more from above than below by 84% [15]. When falling risks are removed from a surface, these illusions disappear [19]. When falling risks are added to a normally perceived surface, these illusions arise [2]. Individuals who most overestimate the risk of falling also most overestimate the length of surfaces from which they could fall [20].

Under ENT, additional exposure to common surfaces should have little effect on adult distance perception unless it substantially alters the likely cost of navigating the surface. Any effects of learning on distance perception should largely be a product of changes to evolutionarily relevant navigation risk. For example, if a person learned to climb more effectively or gained a detailed amount of experience addressing the risks associated with a surface, then distance perception might change–as we see over development. However, most adults have extensive experience walking on surfaces such as flat ground and stairs and so additional experience does not meaningfully alter the likely costs of navigating these common surfaces. There should be little difference in adult distance perception of common surfaces over different levels of exposure.

However, there should be differences in perceived distances where there are differences in navigational risk or cost. Suggested from ENT, observers should overestimate the length of surfaces such as slopes or stairs, especially the vertical aspect of stairs, which accounts for the largest increase in navigational costs via falling risk. Although distance estimates should not change with exposure to any normal set of stairs or horizontal surface (because doing so does not change falling risk), estimates should be very different across stairs and horizontal surfaces because they pose different navigation costs. Previous research clearly outlines such predictions [1], [15], [18], [20], [21].

#### ENT prediction 1

Distance perception should not substantially change across different levels of exposure to a surface.

#### ENT prediction 2

Distance perception should correspond to different levels of evolutionarily relevant risk or cost in navigating the viewed surface wherein high risk corresponds with larger estimates and low risk with smaller estimates.

We tested these three sets of competing predictions in four experiments investigating the effects of exposure and learning on distance perception. Experiment 1 utilized laboratory settings that are typical of distance perception research. Experiment 2 utilized a real-world environment with variable falling risks and participants that had limited, and thus easily quantified, experiences with the estimated surfaces. Experiment 3 also utilized a real-world environment, but with participants who had extensive experience with the estimated surfaces. Finally, Experiment 4 retested the relatively naïve participants from Experiment 2 after they gained substantial experience with the estimated surfaces in order to compare the effects of exposure within individuals. All participants provided written informed consent and the Institutional Review Boards of either the University of Texas or Cal State San Marcos approved all methods.

## Experiment 1

We used typical laboratory distance perception methods in Experiment 1 in order to be able to generalize to the larger body of distance perception research, where highly controlled computer displays are relatively common. The current participants repeatedly estimated distances in a virtual environment presented on a computerized display in a setting with no realistic navigation costs. Within three length conditions, the estimated surfaces were identical in length.

This method was important for understanding the typical context in which most distance perception research exists and to allow generalization to most laboratory based distance perception findings. Such methods did not allow presentation of different navigation costs to participants and so we tested the second set of theoretical predictions in three subsequent experiments. The purpose of this experiment was to test the effect of repeated exposure on distance perception (i.e. Prediction 1) and do so in a way generalizable to most distance perception research.

### Method 1

One hundred and five participants estimated distances in a virtual environment via a head-mounted display under the protocol outlined in Jackson and Cormack, 2010 [19]. Distances appeared on virtual ground and a virtual building, neither of which posed believable falling risks. Participants gave estimates by adjusting the distance between two small dots in the virtual environment via verbal commands to a research assistant until the distance between the dots appeared equal to the length of the estimated surface (i.e. distance matching). Participants gave thirty total distance estimates: five estimates at each of three distances (short [2.72 m], medium [8.37 m], long [14.39 m]) on two orientations (vertical on the building and horizontal on the ground). Participants moved to a different location in the virtual environment after every estimate, which provided additional experience with the estimated surfaces. This process of repeated estimates interspersed with additional exposure to the environment was a benefit of using virtual methods because it emphasizes the effects of exposure, but does not become as tiresome for participants to perform as do similar procedures in the real world.

#### Predictions

We predicted that estimates would become increasingly accurate at each subsequent estimate if exposure affected distance perception (i.e. Learning Hypothesis). This would generate a clear directional trend toward accuracy across each surface estimated. We predicted that estimates would show no such directional trend if exposure did not affect distance perception (i.e. Retinal Image and ENT Hypotheses). Note that laboratory procedures restricted us to testing the second set of predictions concerning differences in navigation risk in three subsequent experiments.

### Results 1

Participants’ distance estimates did not become more accurate over repeated exposure. There was neither a directional trend in distance estimates on any surface, nor meaningful differences across orientation. These data suggest that repeated exposure in a common lab scenario did not affect adult distance perception.

Table 1 displays all estimates and clearly displays no directional change in any of the six estimated surfaces. Although a repeated measures ANOVAs suggested significant differences between estimates within some surfaces (the largest for which *F* (4, 416) = 6.890, *p*<.001), this did not suggest any directional trend, nor trend toward accuracy, for any estimate. After the initial estimate at each orientation and distance, only eleven of the twenty-four subsequent estimates fell closer to the actual distance than the initial estimate. The random likelihood for eleven or more of twenty-four estimates being more accurate than initial estimates is quite high (binomial *p* = .729).

**Table 1:** Experiment 1: Distance Estimates Across Five Laboratory Trials Noting Δ (+,-) From Previous Trial.

Stimulus Orientation		Horizontal			Vertical		
Stimulus Length		Long	Medium	Short	Long	Medium	Short
**Estimate Order**	**First**	15.45	9.34	2.73	15.52	9.14	2.68
	**Second**	15.41(+)	9.42(+)	2.68(−)	15.33(−)	9.23(+)	2.58(−)
	**Third**	15.38(−)	9.42(/)	2.73(+)	15.69(+)	9.31(+)	2.68(+)
	**Fourth**	15.59(+)	9.41(−)	2.67(−)	15.71(+)	9.21(−)	2.62(−)
	**Fifth**	14.99(−)	9.40(−)	2.69(+)	15.18(−)	9.31(+)	2.64(+)
	**Mean**	15.37	9.40	2.70	15.49	9.24	2.64
	**95% CI**	0.24	0.15	0.04	0.20	0.13	0.03

Participants perceived distances nearly identically across horizontal and vertical orientations. Mean estimates differed across orientation by 6 cm (2%) at the short distance, 16 cm (1.0%) at the medium distance, and 12 cm (0.7%) at the long distance.

### Discussion 1

These data suggest that adult environmental distance perception tested with typical laboratory methods and without navigation costs does not become more accurate over repeated exposure. These findings result from a wide range of virtual distances and different surface orientations. The current procedure allowed us to test the relatively rapid initial effects of exposure, rather than exposure over several days, months, or years. Further, this procedure allowed us to limit participant exposure to their lab experience, rather than using surfaces from high-traffic public areas. We found no effect of exposure, in spite of using methods that accentuated exposure by providing repeated estimates of the same surfaces, interspersed with additional exposure to the surfaces between estimates. Laboratory settings facilitated procedural control and provided added emphasis on exposure in ways unavailable from real-world settings.

Interestingly, these data suggest a lack of, not only increased accuracy, but a lack of change in any direction. Participant estimates failed to change substantially in any direction after initial estimates. Although estimates were moderately accurate, they were not perfectly accurate and so it was possible for estimates to improve over repeated exposure, but they did not do so. We predicted from the Learning Hypothesis that participants would learn greater accuracy over time, but we found no support for this prediction. Typical participant distance estimates appeared at first exposure and repeated exposure did little to change this.

Participants perceived surfaces with equivalent falling costs to be equal in length. Although the finding that fixed navigation costs corresponded to fixed distance perception is important, a rigorous test of the underlying idea should include testing distance perception with differences in navigation cost. The invariable navigation costs in this experiment prevented the testing of predictive differences between the Retinal Image and ENT hypotheses. Further, it is essential to conduct real-world experimental procedures in order to generalize to real-world behavior. Laboratory methods allow for limited testing of the effects of normal exposure to a navigable surface.

We addressed these concerns in three additional experiments.

## Experiment 2

### Method 2

Participants estimated the length and height of a set of stairs and equivalent distances on level ground in the real world.

#### Participants

We recruited one hundred and twenty-one undergraduate students on campus for new student orientations who received a cold drink in exchange for their participation. These participants each had a variable, but small, number of experiences with the testing environment and could thus easily remember each of those experiences fairly accurately. This criterion was vital because measurable differences in exposure to the estimated surfaces were imperative for testing our competing predictions. The average student on campus had regularly navigated the experimental setting tens to hundreds of times–the precise number of which would be difficult for them to recall accurately.

#### Stimuli

**Figure 1:**
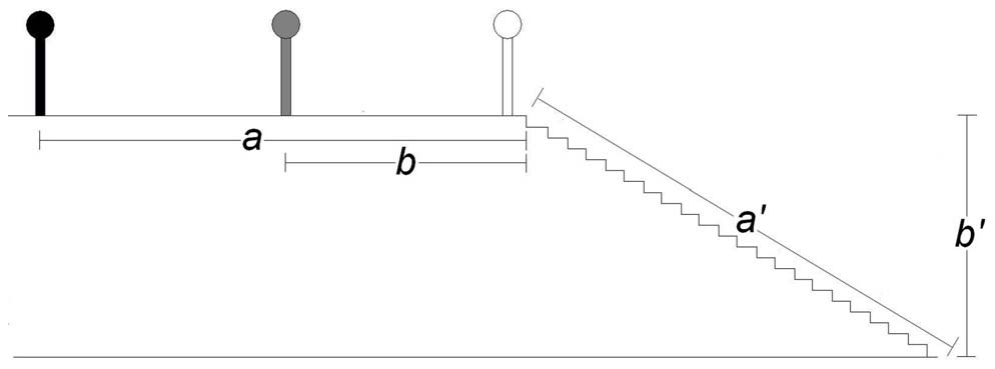
Participant position during Experiments 2–4. The black icon represents participant position while estimating horizontal long (a, 7.11 m), the gray while estimating horizontal short (b, 3.56 m) and the white while estimating stair length (a’, 7.11 m) and stair height (b’, 3.56 m).

We compared participants’ estimates of equivalent distances across two types of surfaces: one that posed falling risks and one that did not pose falling risks. Participants estimated both long (7.11 m) and short (3.56 m) versions of these surfaces, for a total of four estimates (see Figure 1).

The long pair of distance estimates consisted of *stair length* and an equivalent *horizontal long* distance. *Stair length* posed falling costs: it was the distance extending from the edge of the top stair to a marker on the landing at the bottom of the stairs. The *horizontal long* distance was an equal distance on the horizontal ground extending to the top edge of the stairs.

The short pair of distance estimates consisted of *stair height* and an equivalent *horizontal short* distance. *Stair height* posed falling costs: it was the vertical height of the stairs, i.e. the vertical distance from the bottom landing to the top stair. The *horizontal short* distance was an equal distance extending away from the top edge of the stairs on the horizontal ground.

#### Procedure

Participants stood at the top of the stairs when estimating *stair length* and *stair height*. Participants stood a fixed distance away from the stairs when estimating the *horizontal long* and *horizontal short* (7.11 and 3.56 m, respectively).

Participants estimated each of the four distances by manually directing a research assistant (RA) to walk out until the distance from the RA to the participant appeared equal to the distance being estimated. The RA walked out at an angle so that the estimate and the distance being estimated did not appear in the same field of view. Participants could make as many adjustments as they liked in either direction and take as much time as necessary. We used similar distance matching procedures in Experiment 1. Many researchers use this distance matching procedure in realistic outdoor distance estimation for its ability to isolate perceptual processes well [15], [22], [23].

We randomized the order of estimates in three dimensions. First, participants started with either the horizontal estimates or the estimates on the stairs. Second, participants started with either the *length* or *height* within the estimates on the stairs. Third, participants started with either the *long* or the *short* within the horizontal estimates.

After completing all estimates, participants filled out a questionnaire concerning how many times they had been on campus and how many times they had navigated the stairs used in the experiment, as well as items unrelated to the current study.

#### Predictions

The competing predictions applied as follows. We predicted that estimates would be increasingly accurate among individuals with the highest levels of exposure if exposure affected distance perception (i.e. Learning Hypothesis). We predicted that estimates would be equal across differing levels of navigation risk if navigation risk did not affect distance perception (i.e. Learning and Retinal Image Hypotheses). We predicted that estimates would not vary as a function of exposure, but would reflect differences in navigation risk (ENT Hypothesis).

### Results 2

These data replicated the findings of Experiment 1.

Differences in exposure did not predict differences in distance estimation. Number of times navigating the stairs (M ±95% CI = 3.20±1.32) failed to correlate significantly with any distance estimate (the comparison with the lowest *p* value was stair height *r*_120_ = −.135, *p = *.141). Number of times that participants had been on campus (M* = *3.41±0.85) failed to correlate significantly with any distance estimate (the comparison with the lowest *p* value was the horizontal short estimate, *r*_119_ = 0.09, *p* = .313 [one participant did not respond]).

**Figure 2:**
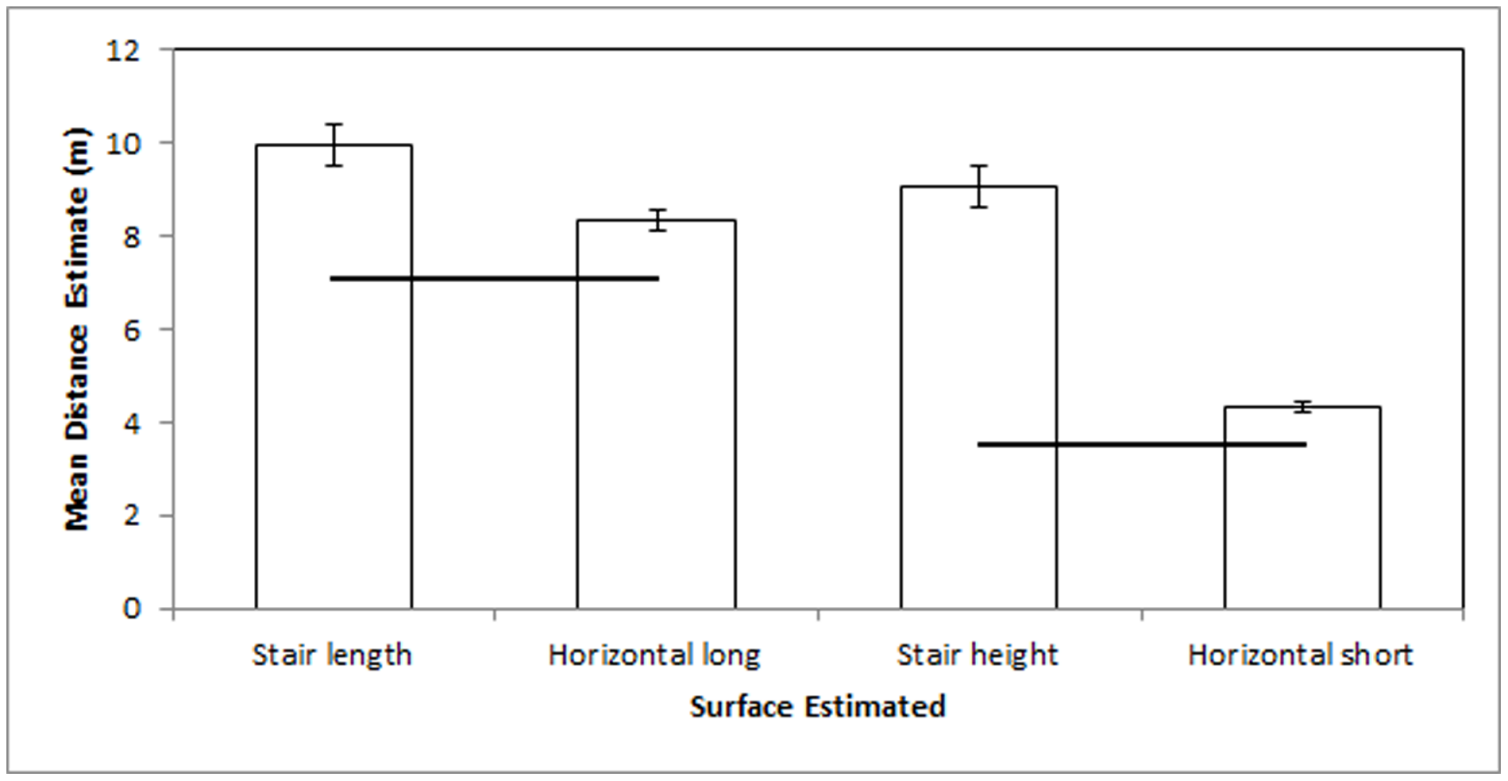
Experiment 2 mean estimates in meters. Black lines indicate actual distances. Error bars represent 95% confidence intervals about the mean.

Differences in falling risks predicted differences in distance estimation (Figure 2). Participants’ estimates slightly exceeded the actual distances and comparison within distance suggests a large effect of falling risk on distance estimate. Stair length estimates (M = 9.98±0.44 m) significantly exceeded by 20% the horizontal long estimates (M* = *8.34±0.21 m), *t*_120_ = 7.48, *p*<.001. Stair height estimates (M* = *9.08±0.45 m) significantly and substantially exceeded by 110% the horizontal short estimates (M* = *4.32±0.10 m), *t*_120_ = 21.89, *p*<.001.

### Discussion 2

Amount of exposure to a real-world surface did not predict differences in distance estimation of that surface, but differences in falling cost predicted distance estimation differences. These data contradict predictions from Learning and Retinal Image Hypotheses, but coincide with predictions from ENT and replicated the findings from the laboratory setting of Experiment 1. Differences in exposure to a common surface would seemingly have little impact on falling risk or other navigational cost and they did not affect distance perception. However, environmental orientation reliably predicted falling risk and it predicted substantial differences in distance estimation.

Experiment 2 participants uniquely possessed a precisely measurable amount of exposure to the surfaces. However, the amount of exposure necessary in order to alter distance perception could consist of higher levels than those experienced by this sample of relatively naïve participants. Furthermore, even if precise exposure measurement would be unavailable in a sample of individuals who navigate the surfaces regularly, such individuals would nonetheless provide a high experience group with which to compare the current sample of low experience participants. For these reasons, we conducted an experiment on participants with high levels of exposure to the testing environment.

## Experiment 3

### Method 3

All stimuli, procedures, and predictions were identical to those in Experiment 2. Participants also made two estimates unrelated to the current study.

#### Participants

One hundred and twenty-eight undergraduate students who regularly attended classes on campus participated in this experiment for course credit. We wanted to test distance perception among participants who had extensive experience with the surfaces in order to determine if high levels of exposure can influence distance estimation differences. This generally more experienced group also importantly provided a comparison with the generally inexperienced participants of Experiment 2.

#### Materials

After participants completed all estimates, they filled out a questionnaire concerning how many times they were on campus in an average week and how many times they navigated the stairs used in the experiment in an average week, similar to Experiment 2. Participants also answered questions unrelated to the current study.

### Results 3

These data replicate the findings of Experiments 1 and 2.

Differences in exposure did not predict differences in distance estimation in this high-exposure sample. The number of times that participants reported using the stairs in an average week (M ±95% CI = 6.29±1.65) failed to correlate significantly with any distance estimate (the comparison with the lowest *p* value was the horizontal short estimate, *r*_127_ = −0.10, *p = *0.26). The number of times that participants reported being on campus in an average week (M = 4.05±.019) failed to correlate significantly with any distance estimate (the comparison with the lowest *p* value was the horizontal long estimate, *r*_127_ = 0.13, *p* = 0.13). Self-reported estimate of the number of times that the participant had ever used the stairs (M = 267.02±278.03) failed to correlate significantly with any distance estimate (the comparison with the lowest *p* value was the stair length estimate, *r*_127_ = −0.05, *p = *0.57), although the accuracy of such an estimate is difficult to determine.

**Figure 3:**
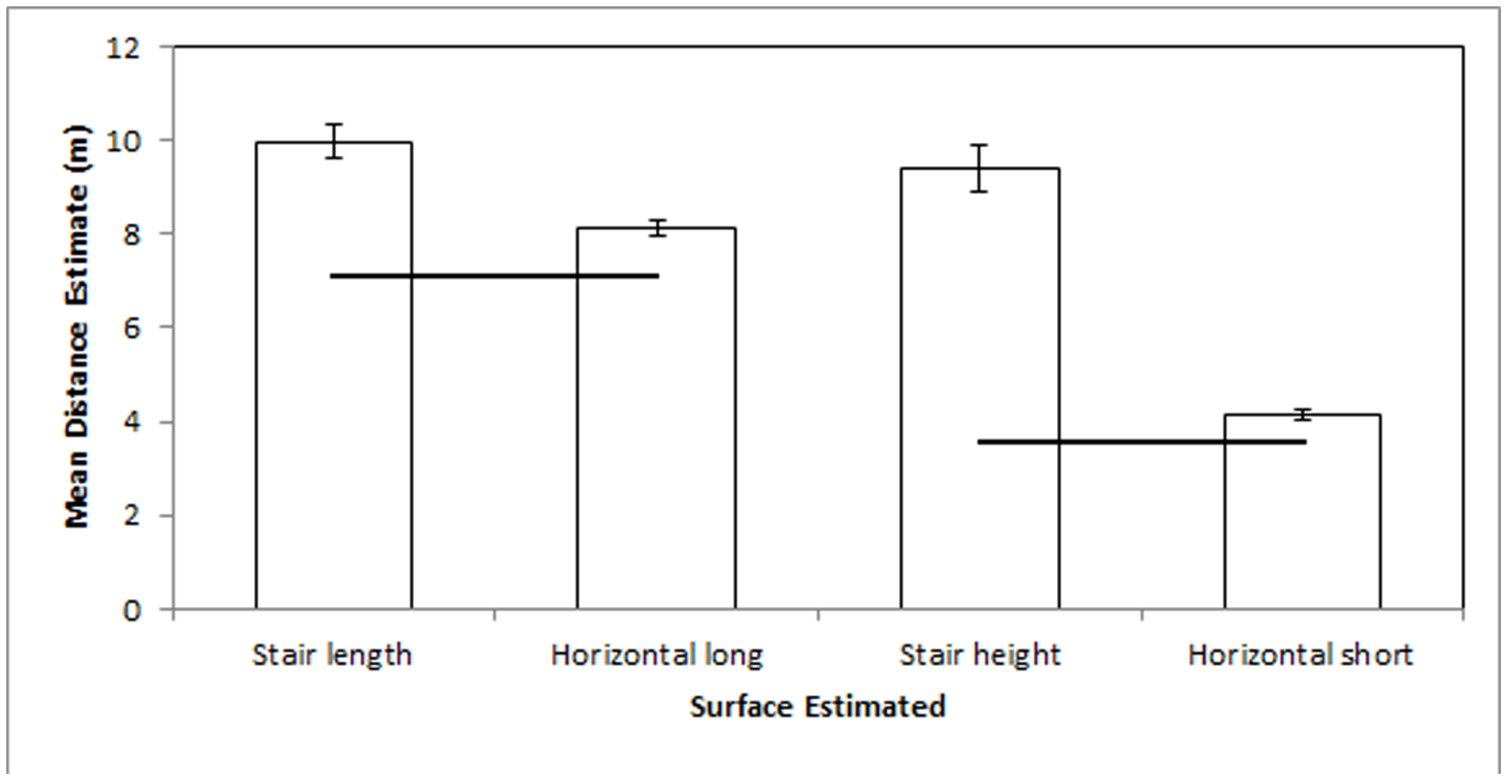
Experiment 3 mean estimates in meters. Black lines indicate actual distances. Error bars represent 95% confidence intervals about the mean.

Differences in falling costs predicted differences in distance estimation in this high-exposure sample (Figure 3). Participants’ estimates slightly exceeded the actual distance across estimates and comparison within distance suggests a large effect of falling risk on distance estimate. Stair length estimates (M* = *9.97±0.37 m) significantly exceeded by 23% the horizontal long estimates (M = 8.11±0.16 m), *t* (127) = 9.68, *p*<.001. Stair height estimates (M = 9.39±0.48 m) significantly and substantially exceeded by 126% the horizontal short estimates (M = 4.15±0.10), *t*_127_ = 21.89, *p*<.001.

#### Results across experiments 2 & 3

Independent samples t-tests (Bonferroni corrected) suggest no meaningful differences in distance estimates between this high exposure sample and the low exposure sample of Experiment 2. Stair length estimates did not differ significantly between the two samples, *t*_247_ = 0.04, *p = *0.97. Horizontal long estimates did not differ significantly between the two samples, *t*_247_ = 1.76, *p* = 0.08. Stair height estimates did not differ significantly between the two samples, *t*_247_ = −0.90, *p = *0.37. Horizontal short estimates differed significantly (*t*_247_ = 2.376, *p* = 0.02), such that low exposure participants from Experiment 2 estimated the surface to be 0.17 m longer on average than the high exposure participants from Experiment 3. This difference comprised less than 4% of the average estimate by the low exposure sample. Such a small difference may appear statistically significant as a byproduct of reduced variability when using relatively large sample sizes, rather than a meaningful difference between the two samples, given that it was very small and contradicted by the results at the other three distances.

### Discussion 3

Differences in exposure did not predict differences in distance estimation within a group of high exposure participants. Further, comparison between groups of high- and low-exposure suggests no clear differences in environmental distance estimation. Horizontal short surface estimates were slightly (17 cm) different across samples and horizontal long estimates were slightly, but not significantly, different. This might suggest estimation improvement from currently obscure conditions and emphasizes the lack of change in vertical and sloped surface estimations. In light of this potential effect across samples, we observed no such effects for any estimate within this sample.

It appeared to be navigation costs, such as likelihood of falling from the estimated surface, that predicted distance estimation differences–both within and between groups of differing levels of exposure. These data correspond with predictions derived from ENT but not predictions derived from the Learning or Retinal Image Hypotheses.

We selected participants for Experiments 2 and 3 that would represent nearly polar ends of an exposure continuum. Comparison across these experiments allowed us the ability to detect distance perception differences available across participants with extreme differences in exposure. Experiment 2 featured participants who had navigated the surface roughly half as many times total as the participants of Experiment 3 navigated the surface in an average week. If exposure altered distance perception, the differences should have been clear between these groups, but no such differences were apparent. It is important to note that our original research question was whether exposure altered distance perception across different levels of exposure *within the same individual*. Comparing the effects of exposure across individuals here allowed us to conjecture that the same effect would occur within individuals. Thus, in order to address our original question directly, we designed a fourth experiment in which we contacted all available participants from Experiment 2 who had matriculated to the university and thus had higher levels of exposure than when we tested them in Experiment 2. We then tested them again in order to determine if their estimates had changed over the course of their increased exposure.

## Experiment 4

### Method 4

All predictions, stimuli, procedures, order, and materials were identical to those in Experiment 3, which were nearly identical to those in Experiment 2.

#### Participants

We contacted all participants from Experiment 2 with the contact information that they had previously provided and asked them to participate in an additional experiment for a ten dollar coffee shop gift card. Thirteen participants had matriculated to the university and agreed to participate in the current experiment, which occurred from three to five months after their initial participation. We did not notify participants that we would contact them in the future during their earlier participation and RAs were blind to this aspect.

### Results 4

These data replicate findings from Experiments 1, 2, and 3.

Differences in exposure over the preceding 3 to 5 months did not predict differences in distance estimation in these participants. The number of times that participants reported using the stairs in an average week (M ±95% CI = 4.77±2.94) failed to correlate significantly with any distance estimate (the comparison with the lowest *p* value was the horizontal short estimate, *r*_12_ = .51, *p = *0.07 [Bonferroni corrected.05 threshold is *p*<.0125]). The number of times that participants reported being on campus in an average week (M = 3.54±0.65) failed to correlate significantly with any distance estimate (the comparison with the lowest *p* value was the horizontal long estimate, *r*_12_ = 0.30, *p* = 0.32).

**Figure 4:**
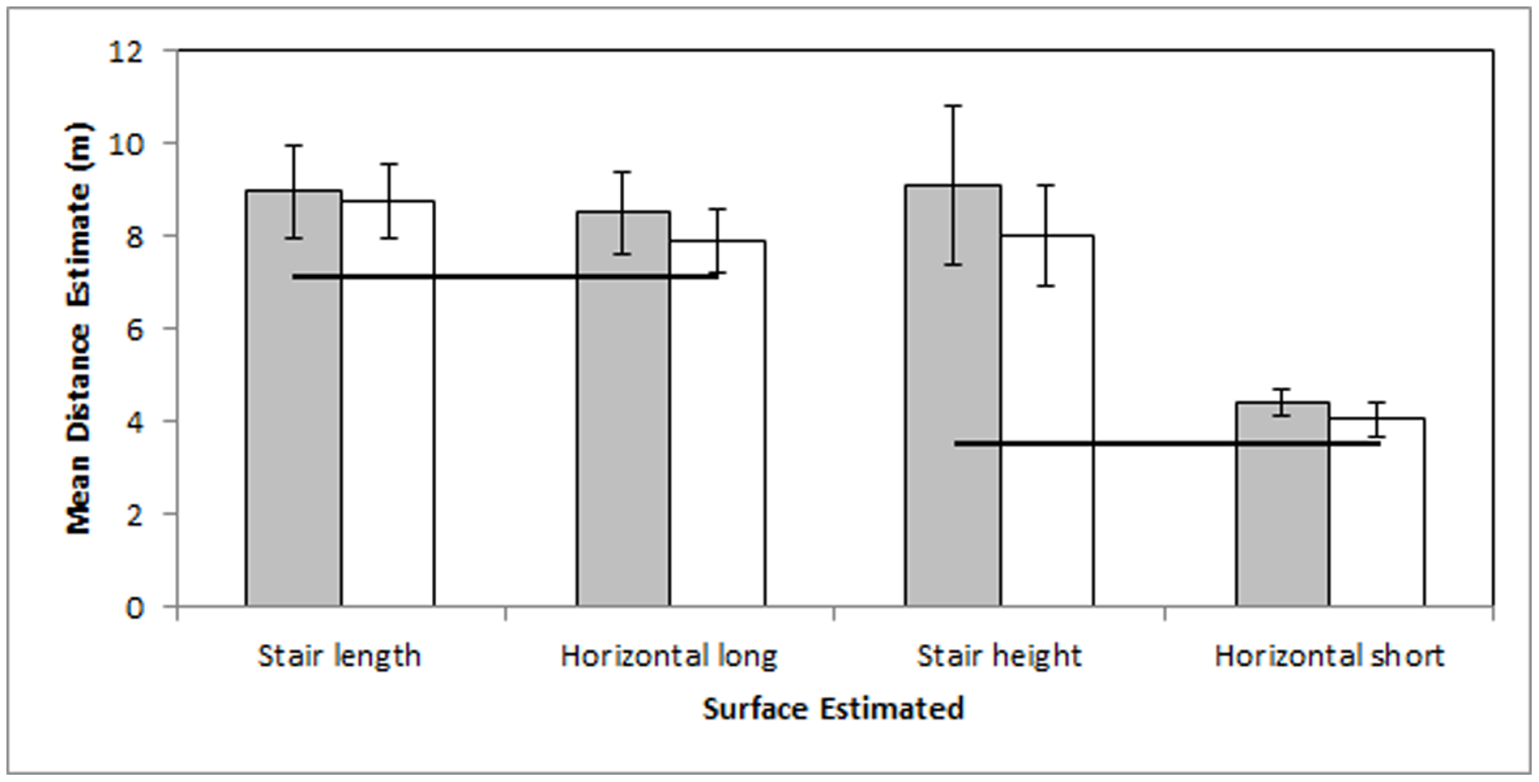
Experiment 4 mean estimates in meters. Estimates by participants at Time 2 (Experiment 4) appear in white and estimates by the same participants at Time 1 (Experiment 2) appear in gray. Black lines indicate actual distances. Error bars represent 95% confidence intervals about the mean.

Differences in falling costs predicted distance perception differences in these participants (see Figure 4). Participants’ estimates slightly exceeded the actual distance across estimates and comparison within distance suggests a large effect of falling risk on distance estimate. Stair length estimates (M = 8.76±0.82 m) significantly exceeded by 11% the horizontal long estimates (M = 7.89±0.71 m), *t*_12_ = 2.68, *p = *0.02. Stair height estimates (M = 8.00±1.11 m) significantly and substantially exceeded by 98% the horizontal short estimates (M = 4.04±0.35 m), *t*_12_ = 8.47, *p*<.001.

#### Results across experiments 2 & 4

Exposure to the estimated surfaces changed in this sample, but estimates of the surfaces did not change significantly.

Exposure to the estimated surfaces drastically increased. These participants reported an average of 5.35±5.07 total times ever taking the stairs in Experiment 2, but reported taking the stairs 4.77±2.94 times per average week over the preceding three to five months in Experiment 4. The total number of times that participants reported ever being on campus at Experiment 2 averaged 2.69±1.73, while the average number of times that participants reported being on campus in an average week over the preceding three to five months at Experiment 4 was 3.54±0.65.

Paired samples *t*-tests suggested no significant differences in any distance estimates between Experiment 2 and Experiment 4 (the comparison with the lowest *p* value was the horizontal short estimate, *t*_12_ = 2.11, *p = *.06 [Bonferroni corrected.05 threshold is *p*<.0125]).

### Discussion 4

Additional exposure did not predict distance estimation differences within individuals. Falling cost differences predicted distance estimation differences across all levels of experience tested within individuals. These data contradict predictions from Learning and Retinal Image hypotheses, but coincide with predictions from ENT.

A strong predictor of both falling costs and distance estimation investigated here was surface orientation, as predicted under ENT. Passive exposure differences within the same adult on the common surfaces tested here would do little to change that person’s risk of falling or suffering other navigational costs and exposure did not significantly affect distance estimates. Although we found no significant effect, we were interested in any effect of exposure on distance estimation. On average, participants’ estimates were slightly more accurate in Experiment 4 than Experiment 2 across surfaces. Compared to Experiment 2 estimates, Experiment 4 estimates were more accurate by 2.17% in the stair length, 7.13% in the horizontal long, 11.79% in the stair height, and 8.56% in the horizontal short estimates. The binomial probability for an increase in accuracy across all four estimates equals.0625. If this increased accuracy was genuine, the size of the difference (2–12%) was nonetheless far smaller than the effect of environmental orientation (11–98%).

Although some small effect of exposure that increased distance perception accuracy seems plausible, it only occurred in Experiment 4, in which we tested the same individuals twice. Given that similar effects did not occur under conditions of relatively rapid sequential estimates within subjects (Experiment 1), nor similar estimates between-subjects (i.e. Experiments 2 & 3), a change in procedural accuracy seems more plausible and more supported by previous research than a change in underlying distance perception [13], [14].

## General Discussion

We observed a very strong effect of apparent navigation costs on distance estimation, but found little, if any, effect of exposure. This occurred in adults in everyday environments and laboratory settings across four experiments investigating exposure differences within and between individuals. Not only did exposure pose no clear main effect on estimation, but there was also no apparent interaction of exposure and the effect of navigation costs on distance estimates.

Major areas of behavioral science predicted clear, but contradictory, effects of navigational costs and exposure. Some vision science research nearly exclusively relies upon understanding the retinal image in order to determine distances in the environment. These data fail to support such claims. However, this should not be interpreted to suggest that retinal image is unimportant. To the contrary, retinal image is irreplaceable in visual distance perception. These data merely outline that retinal image alone does not determine adult distance perception. Human post-retinal processing can entirely reverse the distances apparent in the retinal image, as seen in these data.

We predicted from the Learning Hypothesis that participants’ estimates would become increasingly accurate over time. These data fail to support such a prediction. Even if one were to suggest from a learning approach that humans estimate stairs inaccurately because we navigate them less than we navigate flat ground, it would still fail to explain the observed directional overestimation. Participants did not display simple inaccuracy for the falling risk surfaces; inaccuracy would be over- and under-estimates, instead of the directional overestimates observed here. From ENT, we did not predict simple overestimation of all surfaces–only the surfaces with evolutionarily relevant navigation costs. These experiments supported ENT predictions that differed across scenarios: surfaces without different navigation costs should appear equal (Experiment 1) and did so. Surfaces with different navigation risks should appear different in length (Experiments 2, 3, & 4) and did so.

It is important to note that slight deviations from the intended estimate are common in real-world distance estimation experiments [22], [24]–[27]. Indeed, participants in the current experiments at least slightly overestimated all distances, including those without falling costs. However, this effect was small (11–21% of the actual distance) in comparison to that of the falling cost surfaces (23–164% of the actual distance). Slight deviations from accuracy are likely common in real-world vision experiments because the number and variability of features present in the stimuli are effectively infinite. One way to avoid drawing incorrect conclusions in light of these slight deviations is to be particularly cautious of small effects.

There was potentially weak indirect support for a small increase in accuracy in one component of the fourth experiment. This may have stemmed from increased participant facility with the experimental procedures or increased attention directed at the research site after completing the previous experiment. Neither of these reasons generalizes to increased perceptual accuracy stemming from passive exposure to common surfaces, which was the question under investigation. If the potential accuracy increase due to exposure was genuine, it was very small–far smaller than the effect of environmental orientation.

We suggest from ENT that distance estimation accuracy could, in fact, increase significantly due to exposure, but that this would be a byproduct of changes in ability to address navigation costs. Such an effect might increase accuracy in ways that fall outside of the current investigation. For example, exposure to novel, instead of common, surfaces might result in greater increases in accuracy of the respective surface. We measured exposure to common surfaces in the current investigation because we wanted to be able to generalize to the majority of adult human navigation. We also wanted to avoid evolutionarily novel navigation scenarios, but novel scenarios seem a likely candidate for rapid increases in what would likely be a low base level of accuracy. Similarly, the effects of exposure on accuracy likely change over lifespan, with a large effect during early formational ages when many navigational scenarios are effectively novel [28]. Further, differences in previous navigational experiences could produce differences in distance estimation. Additionally, active and deep processing might increase accuracy more than passive exposure. If an observer attended to the distance during exposure and actively sought ways to verify the distance, it might change distance estimates by informing the observer of likely navigation costs and responses to those costs. Such an effect may have occurred in Experiment 4. Similarly, a learning period during which the observer receives feedback, rather than simply exposure, would be a likely candidate for increased accuracy of *estimate*; however, whether or not such a process would change the underlying *perception* is an important consideration. Ultimately, all of the above exceptions deviate from the original research question–whether or not common exposure affects distance perception accuracy in adults–that the current data failed to support.

The current data are consistent with the ENT prediction that environmental orientations corresponding to navigational costs predict distance estimation. This adds to a growing body of research that investigates how evolution by natural selection may have acted on environmental perception [29]–[31]. Specifically, ENT researchers have specified distance perception as one proximal mechanism that can alter navigational choice to reflect navigational costs [1], [2], [15], [18]–[21]. The current participants overestimated surfaces in ways that reflected navigational cost. Participants overestimated the highest falling cost surface (stair height) by five to nine times as much as they overestimated the moderate falling cost surface (stair length). The predictions under ENT were the only predictions consistent with these data.

Although ENT predictions contradicted the predictions from the other research approaches, ENT is not mutually exclusive with appropriate application of the mechanisms proposed under these approaches. Certainly retinal information and learning are two essential mechanisms in the evolution of vision. However, without placing these two mechanisms in an evolutionarily plausible context, they failed to make supportable predictions or demonstrate effects of the magnitude demonstrated by mechanisms of greater evolutionary consequence.

High human capacity for learning might initially appear to suggest that exposure should strongly shape distance perception, even in adults and especially when the initial estimates were far from accurate. However, the current data suggest relatively low plasticity in this dimension of perception. Such distance perception constancy over experience might seem somewhat detrimental or nonadaptive, which is certainly possible. However, distance perception constancy provides a stable signal that allows a large range of behaviors to reflect costs accurately. Falling costs are likely easily determined quickly from little more than visual information on length and orientation of a surface, and so repeated exposure likely provides little additional information in order for distance estimation to reflect navigational cost well. An adaptive response to selection from the costs of falling over evolutionary time may have been to produce distance estimates that reflect falling costs well initially (as observed), which eliminates the need for reevaluation of distances based upon degree of exposure (as observed). The stable signal to navigation costs provided by such a mechanism would allow many downstream processes to function without the exponential increase in processing complexity required from constantly reassessing navigation risks. Such stability of navigation risk signaling may be the very factor that allows other behavioral and cognitive adaptations to account for navigational costs.

The current findings relate to pervasive selection pressures across domains. The costs of navigation precede the costs of most behaviors, including essential behaviors such as mating, parenting, and food acquisition. Our data may suggest that navigational mechanisms are shaped such that normal experience with one’s environment reflects navigation costs well and repeated exposure does little to change this.

## References

[1] Jackson R (2005) Falling towards a theory of the vertical-horizontal illusion. Studies in Perception and Action 8: 241–244.

[2] Jackson R, Willey C (2011) Evolved navigation theory and horizontal visual illusions. Cognition 119: 288–294 doi:10.1016/j.cognition.2010.11.003 21115176 10.1016/j.cognition.2010.11.003

[3] Gilinsky A (1951) Perceived size and distance in visual space. Psychol Rev 58: 460–482. 14900306 10.1037/h0061505

[4] Kudoh N (2005) Dissociation between visual perception of allocentric distance and visually directed walking of its extent. Perception 34: 1399–1416 doi: 10.1068/p5444 16355744 10.1068/p5444

[5] Loomis J, Da Silva J, Fujita N, Fukusima S (1992) Visual space perception and visually directed action. J Exp Psychol 18: 906–921. 10.1037//0096-1523.18.4.906 1431754

[6] Collins J (1976) Distance perception as a function of age. Aust J Psychol 28: 109–113.

[7] Adolph K (2000) Specificity of learning: Why infants fall over a veritable cliff. Psychol Sci 11: 290–295. 11273387 10.1111/1467-9280.00258

[8] Campos J, Bertenthal, Kermoian R (1992) Early experience and emotional development: The emergence of wariness of heights. Psychol Sci 3: 61–64.

[9] Harway N (1963) Judgment of distance in children and adults. J Exp Psychol 65: 385–390. 13960805 10.1037/h0046363

[10] Pietrzak R, Maruff P, Mayes L, Roman S, Sosa J, et al (2008) An examination of the construct validity and factor structure of the Groton Maze Learning Test, a new measure of spatial working memory, learning efficiency, and error monitoring. Arch Clin Neurosycho 23: 433–445. 10.1016/j.acn.2008.03.002 18448309

[11] Rieser J (2000) Concepts about the causes of development: Travel, visual experience, and the development of dynamic spatial orientation. Infancy 1: 231–238. 10.1207/S15327078IN0102_4 32680293

[12] Witherington D, Campos J, Anderson D, Lejeune L, Seah E (2005) Avoidance of heights on the visual cliff in newly walking infants. Infancy 7: 285–298. 10.1207/s15327078in0703_4 33430562

[13] Garling T, Lindberg E, Mantyla T (1983) Orientation in buildings; Effects of familiarity, visual access, and orientation aids. J Appl Psychol 68: 177–186. 6826491

[14] van Asselen M, Fritschy E, Postma A (2006) The influence of intentional and incidental learning on acquiring spatial knowledge during navigation. Psychol Res 70: 151–156. 15844006 10.1007/s00426-004-0199-0

[15] Jackson R, Cormack L (2007) Evolved navigation theory and the descent illusion. Percept Psychophys 69: 353–362. 17672423 10.3758/bf03193756

[16] Jackson R (2013) Preference for the nearer of otherwise equivalent navigational goals quantifies behavioral motivation and natural selection. PLoS One 8: 1–4 doi: 10.1371/journal.pone.005 10.1371/journal.pone.0054725 PMC3552966 23355894

[17] DeCamp J (1920) Relative distances as a factor in the white rat’s selection of a path. Psychobio 2: 245–253 doi: 10.1037/h0075411

[18] Jackson R, Cormack L (2008) Evolved navigation theory and the environmental vertical illusion. Evol Hum Behav 29: 299–304.

[19] Jackson R, Cormack L (2010) Reducing the presence of navigation risk eliminates strong environmental illusions. J Vis 10: 1–8 doi:10.1167/10.5.9 10.1167/10.5.9 20616118

[20] Jackson R (2009) Individual differences in distance perception. Proc Roy Soc B-Biol Sci 276: 1665–1669 doi: 10.1098/rspb.2009.0004 10.1098/rspb.2009.0004 PMC2660999 19324829

[21] Jackson R (2007) Evolved navigation theory and the environmental vertical illusion. Doctoral dissertation, University of Texas, Austin.

[22] Chapanis A, Mankin D (1967) The vertical-horizontal illusion in a visually-rich environment. Percept Psychophys 2: 249–255.

[23] Higashiyama A (1996) Horizontal and vertical distance perception: The discorded-orientation theory. Percept Psychophys 58: 259–270. 8838168 10.3758/bf03211879

[24] Bridgeman B, Hoover M (2008) Processing spatial layout by perception and sensorimotor interaction. Q J Exp Psychol 61: 851–859. 10.1080/17470210701623712 18470816

[25] Hansen B, Essock E (2004) A horizontal bias in human visual processing of orientation and its correspondence to the structural components of natural scenes. J Vis 4: 1044–1060. 15669910 10.1167/4.12.5

[26] Mankin D (1969) The influence of perceptual anchors and visual noise on the vertical horizontal illusion. Percept Psychophys 5: 149–154.

[27] Taylor M (1961) Effect of anchoring and distance perception on the reproduction of forms. Percept Mot Skills 12: 203–230.

[28] Gibson E, Walk R (1960) The “visual cliff.”. Sci Am 202: 64–71. 13827949

[29] Bridgeman B (2005) Action planning supplements mirror systems in language evolution. Behav Brain Sci 28: 129–130.

[30] Gaulin S, Hoffman H (1988) Evolution and development of sex differences in spatial ability. In L. Betzig, M. Borgerhoff Mulder, & P. Turke (Eds.), Human reproductive behavior: A Darwinian perspective (pp. 129–152). Cambridge.

[31] New J, Krasnow M, Truxaw D, Gaulin S (2007) Spatial adaptations for plant foraging: Women excel and calories count. Proc Roy Soc B-Biol Sci 274: 2679–2684. 10.1098/rspb.2007.0826 PMC2279214 17711835

